# Supplementary figures and images for: Meta-analysis of variation suggests that embracing variability improves both replicability and generalizability in preclinical research
Source: PLoS Biol. 2021 May 19;19(5):e3001009. doi: 10.1371/journal.pbio.3001009 (PMC8168858; doi:10.1371/journal.pbio.3001009)

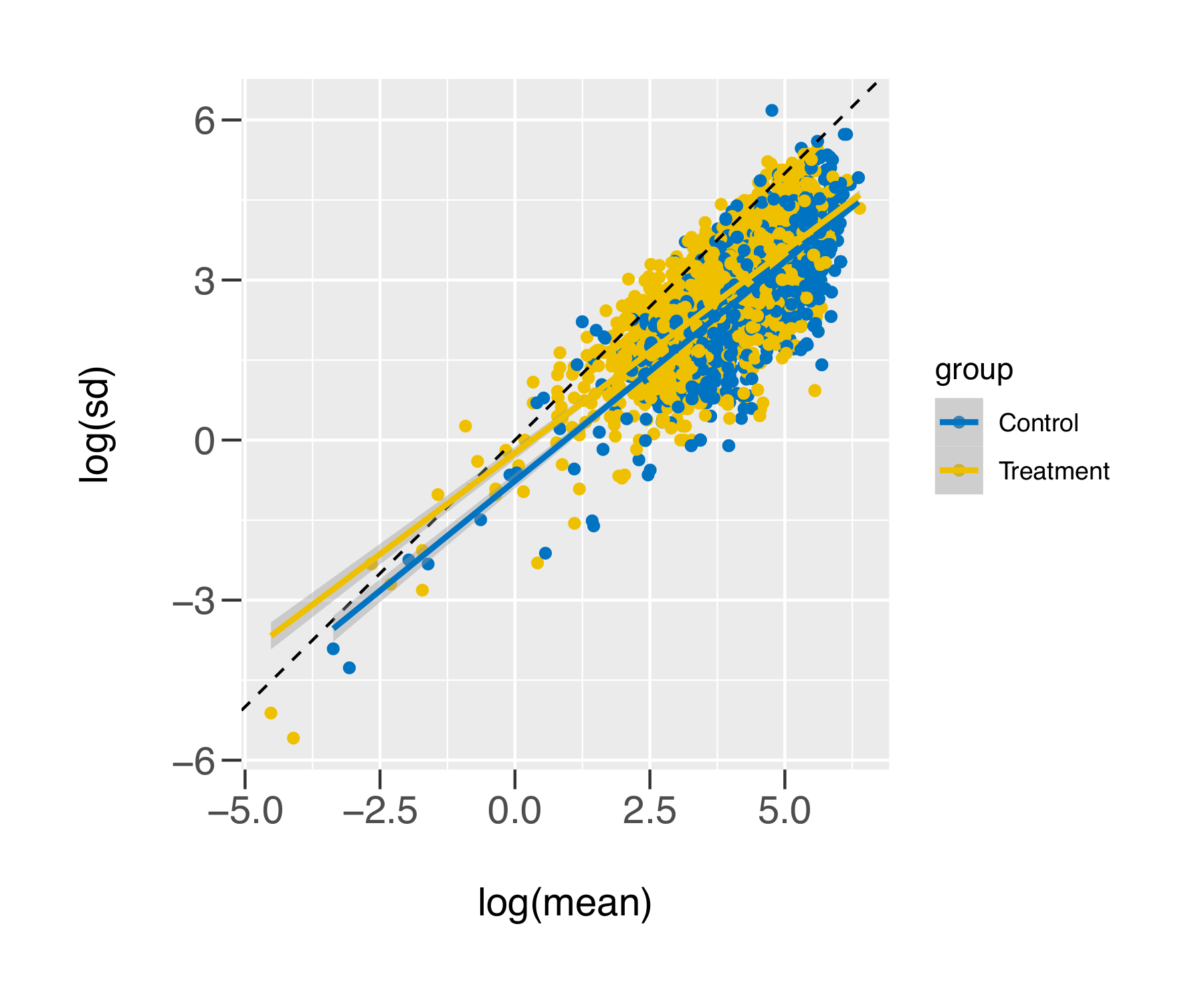

Supplement: S1 Fig — Point estimates for control (blue) and treatment (yellow) groups are provided. Slopes and 95% CIs from linear regressions for control (0.822, 0.791 to 0.854) and treatment (0.758, 0.728 to 0.788) rat groups, respectively, are shown. The data underlying this figure can be found at https://doi.org/10.6084/m9.figshare.14527317.v4. CI, credible interval. (TIF) [file pbio.3001009.s001.tif]

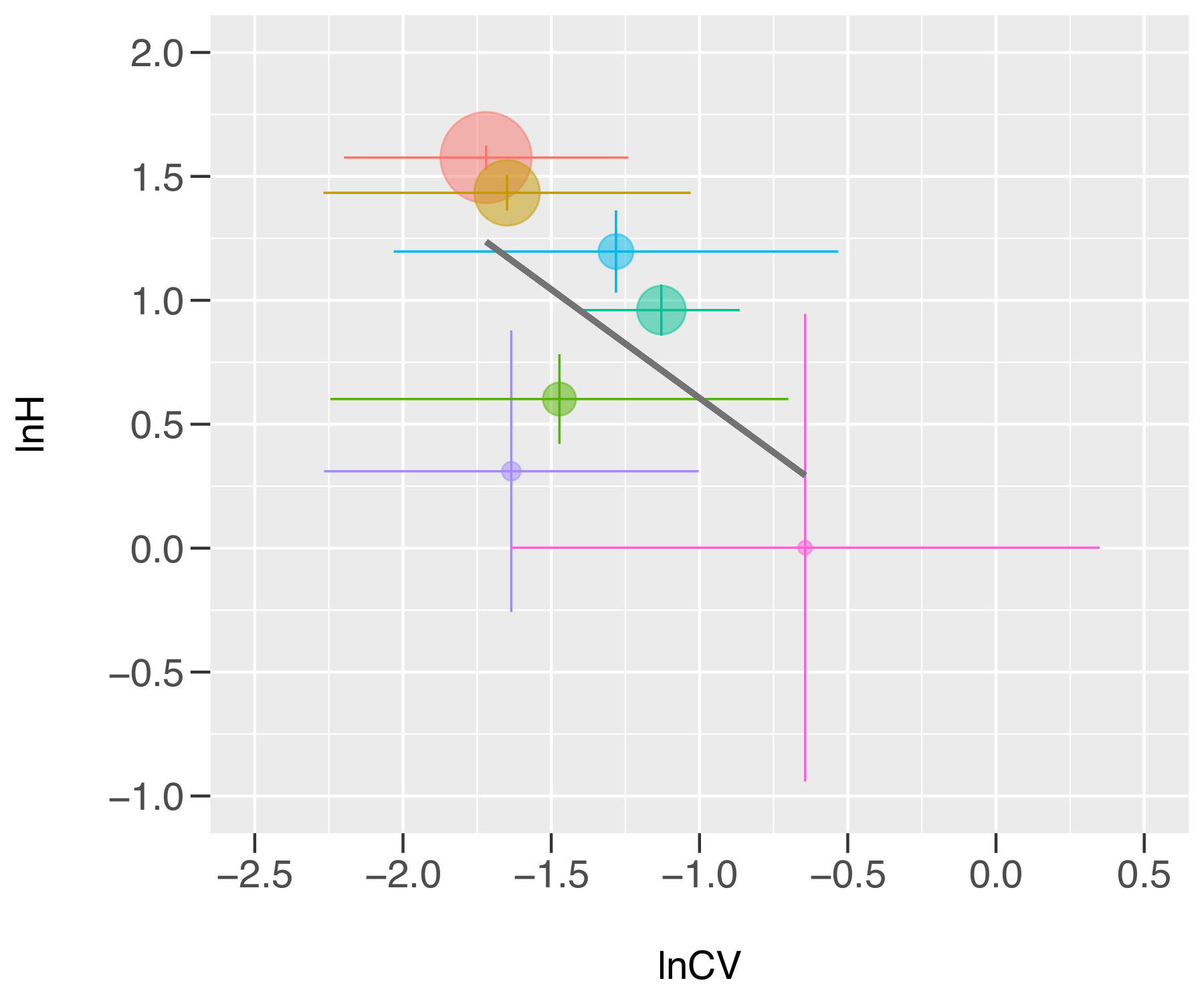

Supplement: S2 Fig — The mean slope of the relationship (slope = −0.876, −2.047 to 0.295) from the MLMR model is shown by the gray line. Circles represent estimates for each occlusion method and solid lines their 95% CIs obtained from multilevel regression (MLMR) models. Each color represents a different occlusion method, and circle sizes represent the number of effect sizes available to estimate lnH for each occlusion method. From the highest to lowest lnH estimates: orange = Filament [N = 973]; yellow = Mechanical/direct [N = 438]; blue = Endothelin injection [N = 76]; turquoise = Emboli/clot [N = 201]; green = Photothrombotic [N = 64]; purple = Collagenase injection [N = 8]; pink = spontaneous [N = 4]. The data underlying this figure can be found at https://doi.org/10.6084/m9.figshare.14527317.v4. CI, credible interval; lnCV, log coefficient of variation; MLMR, multilevel meta-regression. (TIF) [file pbio.3001009.s002.tif]

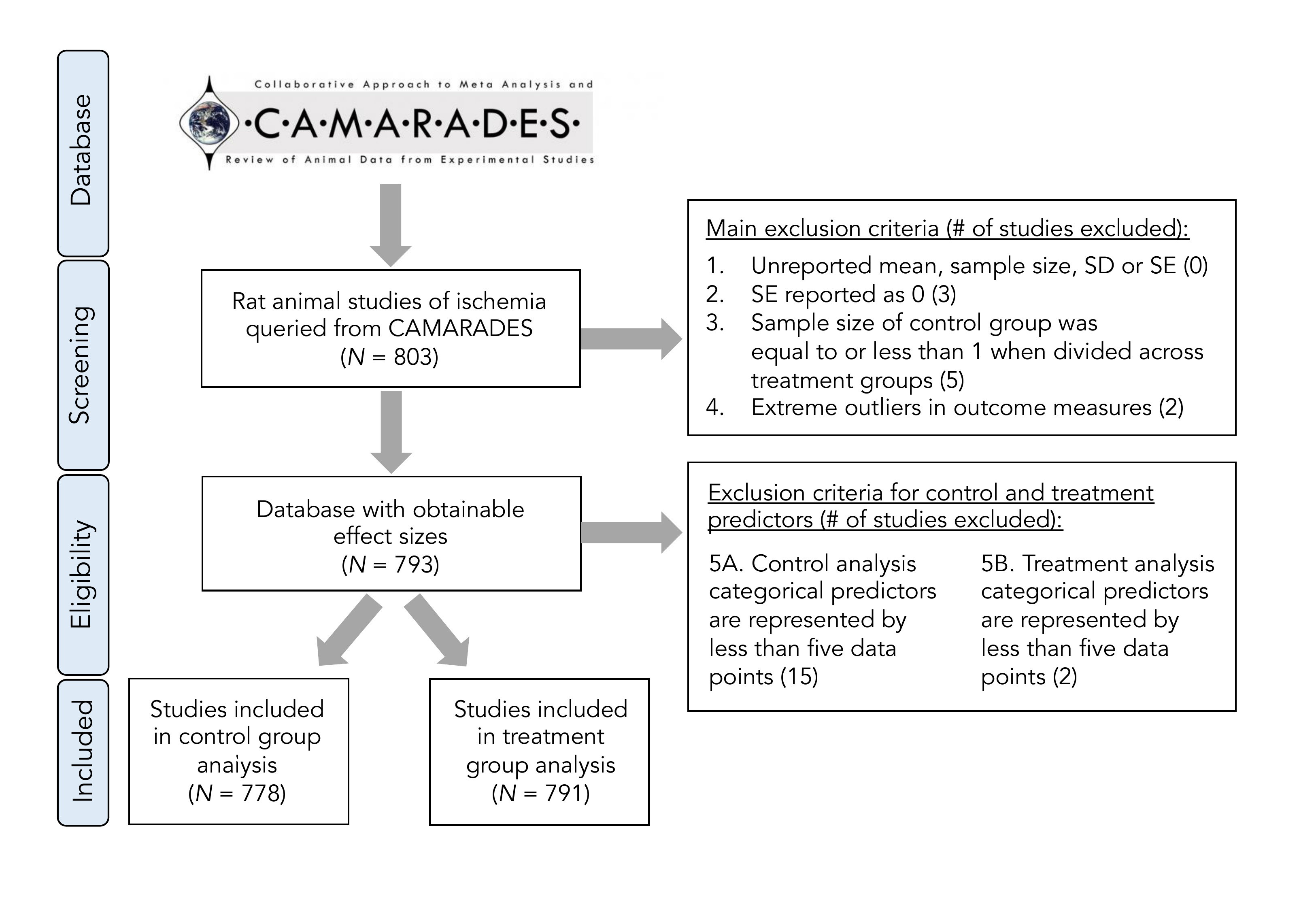

Supplement: S3 Fig — PRISMA, Preferred Reporting Items for Systematic Reviews and Meta-Analyses. (TIF) [file pbio.3001009.s003.tif]

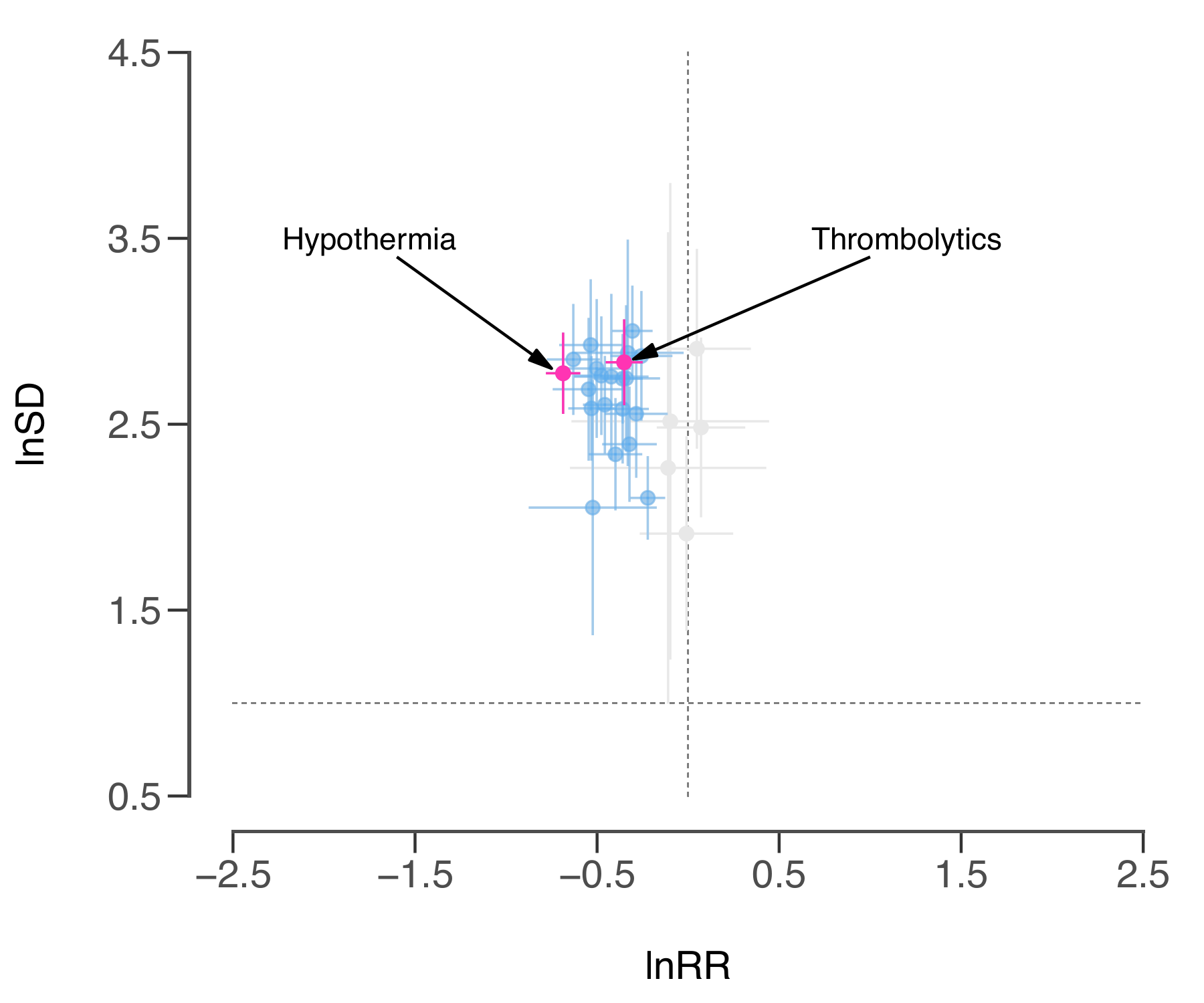

Supplement: S4 Fig — Estimates (circles) represent unconditional (marginalized), treatment-specific means (lnRR), variability (lnSD), and their 95% CIs (solid lines). We reveal differences in variability across treatment groups, with treatments that significantly reduce infarct volume and increase interindividual variability (positive lnSD) highlighted blue. The effects of hypothermia and thrombolytics (the latter of which include the only regulatory-approved treatment) are highlighted in pink. The data underlying this figure can be found at https://doi.org/10.6084/m9.figshare.14527317.v4. CI, credible interval; lnRR, log response ratio; lnSD, log standard variation. (TIF) [file pbio.3001009.s004.tif]

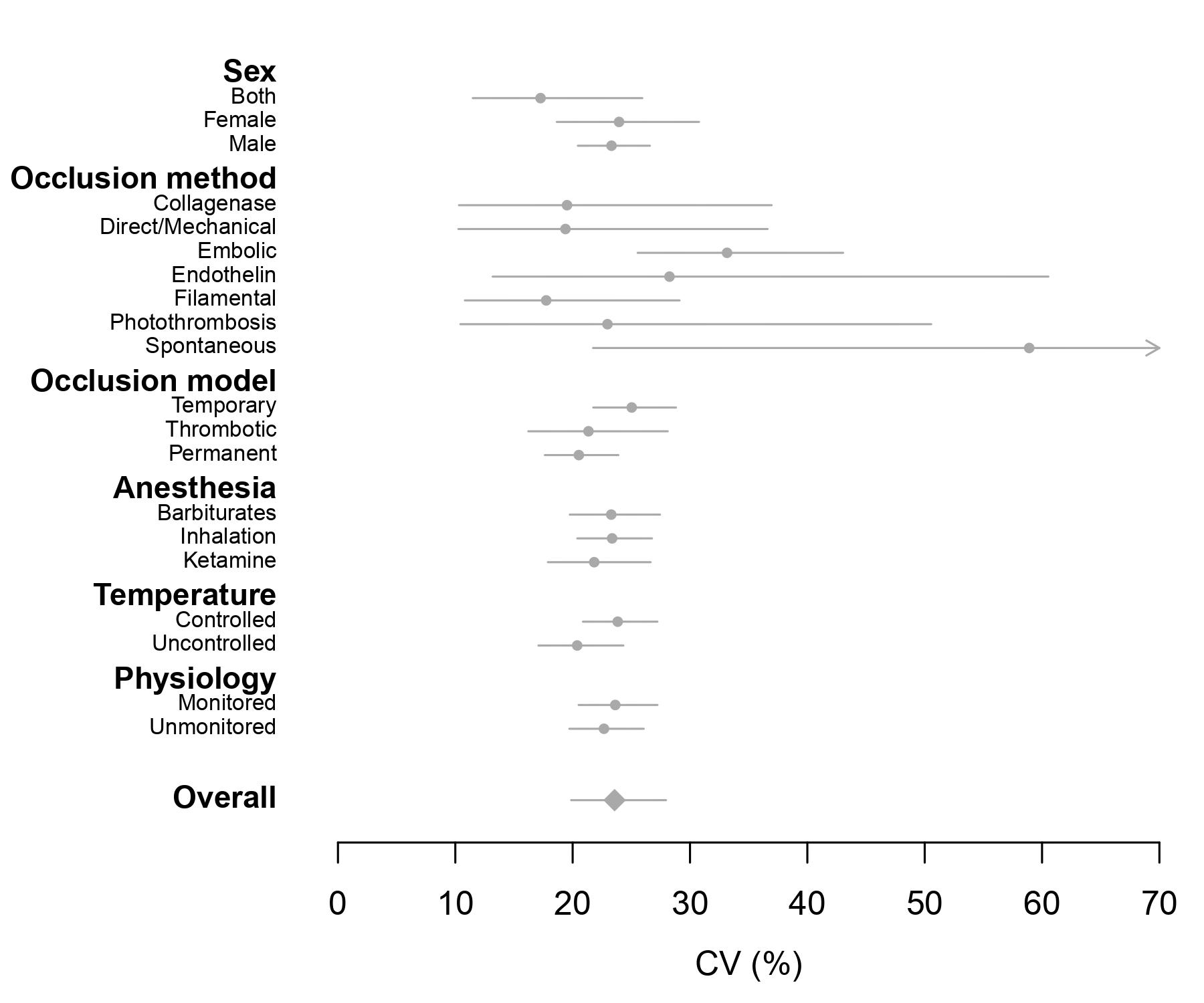

Supplement: S5 Fig — Mean estimates of unconditional (marginalized), group-specific coefficients of variation (%) are indicated as gray circles. Moreover, 95% CIs are shown as gray lines and are asymmetric due to back-transformation of log coefficient of variation (lnCV) to the natural scale. The data underlying this figure can be found at https://doi.org/10.6084/m9.figshare.14527317.v4. CI, credible interval; lnCV, log coefficient of variation; MLMR, multilevel meta-regression. (TIF) [file pbio.3001009.s005.tif]

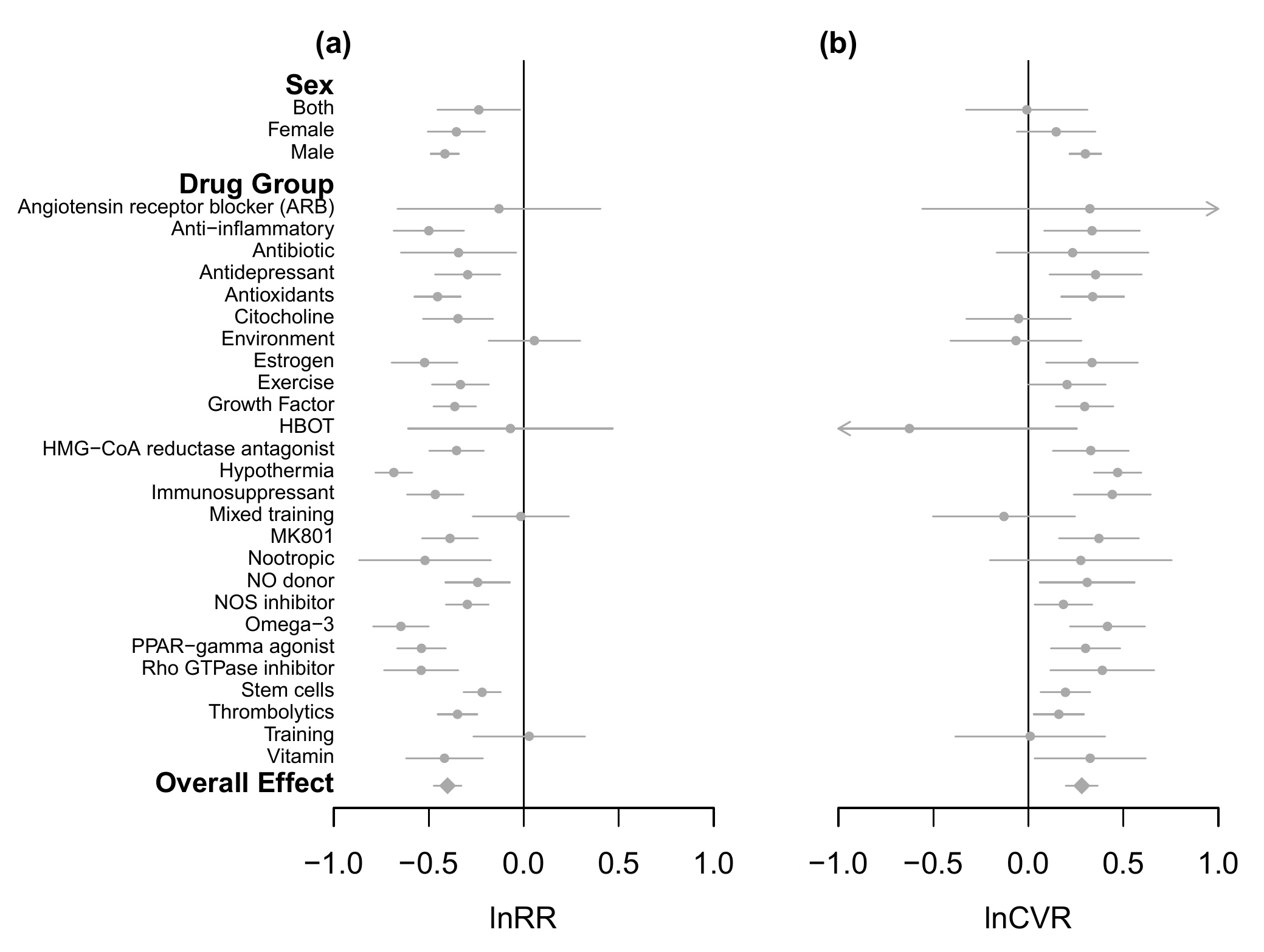

Supplement: S6 Fig — Sensitivity model (a) lnRR and (b) lnCVR estimates from multilevel regression (MLMR) of infarct volume in treatment/control groups. Mean estimates of unconditional (marginalized), group-specific effects are shown as gray circles, and 95% CIs are shown as gray lines. Negative lnRR estimates indicate that mean infarct volume is smaller in experimental versus control rats. Negative lnCVR estimates show that interindividual variability in infarct volume is smaller in experimental versus control rats. The data underlying this figure can be found at https://doi.org/10.6084/m9.figshare.14527317.v4. CI, credible interval; lnCVR, log coefficient of variation ratio; lnRR, log response ratio; MLMR, multilevel meta-regression. (TIF) [file pbio.3001009.s006.tif]

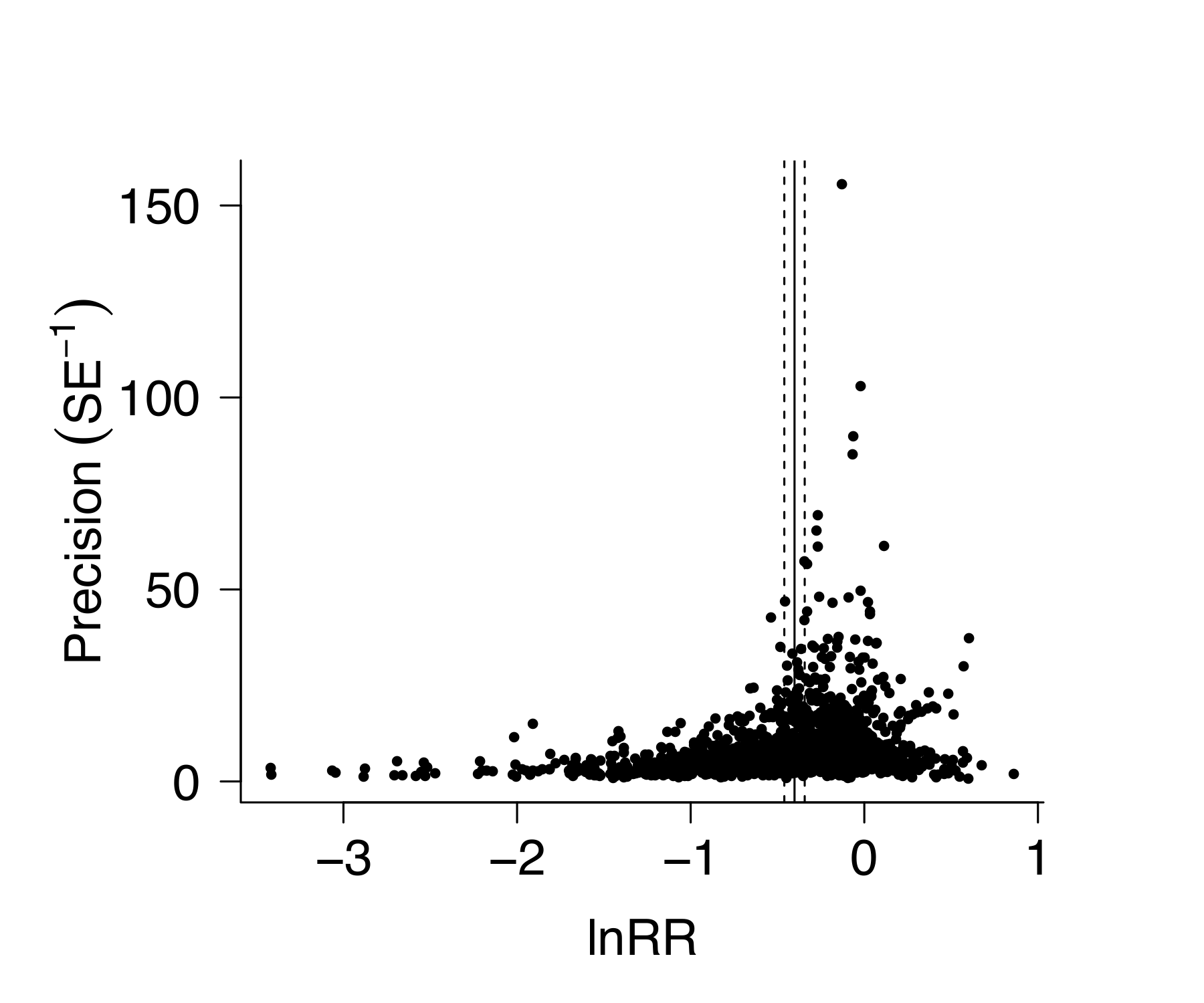

Supplement: S7 Fig — Raw effect sizes are plotted against their precision (inverse of the square root of standard error). MLMA model predicted mean effect size (solid vertical line), and its 95% CI (dashed lines) are shown. The data underlying this figure can be found at https://doi.org/10.6084/m9.figshare.14527317.v4. CI, credible interval; lnRR, log response ratio; MLMA, multilevel meta-analysis. (TIF) [file pbio.3001009.s007.tif]
