# Supplementary material for: Meta-analysis of variation suggests that embracing variability improves both replicability and generalizability in preclinical research
Source: PLoS Biol. 2021 May 19;19(5):e3001009. doi: 10.1371/journal.pbio.3001009 (PMC8168858; doi:10.1371/journal.pbio.3001009)
Supplement: S1 Table — Continuous predictors were Z-transformed prior to model fitting. lnCV, log coefficient of variation; MLMR, multilevel meta-regression. (DOCX) [file pbio.3001009.s008.docx]

**S1 Table.** Unconditional (marginalized) estimates and 95% credible intervals for lnCV, obtained from multi-level regression (MLMR) models of control group infarct volume. Continuous predictors were Z-transformed prior to model fitting.

| Parameters | $lnCV (\beta)$ | LCI | UCI |
| --- | --- | --- | --- |
| Sex _BOTH_ | -1.757 | -2.150 | -1.364 |
| Sex _FEMALE_ | -1.429 | -1.670 | -1.188 |
| Sex _MALE_ | -1.450 | -1.561 | -1.339 |
| InductionMethod _COLLAGENASE_ | -1.635 | -2.263 | -1.007 |
| InductionMethod _EMBOLIC_ | -1.129 | -1.389 | -0.869 |
| InductionMethod _ENDOTHELIN_ | -1.282 | -2.027 | -0.536 |
| InductionMethod _FILAMENTAL_ | -1.720 | -2.195 | -1.244 |
| InductionMethod _DIRECT/MECHANICAL_ | -1.649 | -2.264 | -1.034 |
| InductionMethod _PHOTOTHROMBOSIS_ | -1.472 | -2.241 | -0.704 |
| InductionMethod _SPONTANEOUS_ | -0.644 | -1.633 | 0.345 |
| IschaemiaModel _PERMANENT_ | -1.583 | -1.719 | -1.448 |
| IschaemiaModel _TEMPORARY_ | -1.377 | -1.500 | -1.255 |
| IschaemiaModel _THROMBOTIC_ | -1.533 | -1.813 | -1.252 |
| Anesthesia _KETAMINE_ | -1.496 | -1.687 | -1.304 |
| Anesthesia _INHALATION_ | -1.454 | -1.570 | -1.338 |
| Anesthesia _BARBITURATES_ | -1.445 | -1.592 | -1.298 |
| TemperatureControl _NO_ | -1.605 | -1.764 | -1.445 |
| TemperatureControl _YES_ | -1.424 | -1.536 | -1.311 |
| PhysiologyMonitored _NO_ | -1.475 | -1.595 | -1.355 |
| PhysiologyMonitored _YES_ | -1.441 | -1.563 | -1.318 |
| AssessTime | -1.404 | -1.521 | -1.288 |
| MidWeight | -1.366 | -1.486 | -1.245 |
