# Supplementary material for: Meta-analysis of variation suggests that embracing variability improves both replicability and generalizability in preclinical research
Source: PLoS Biol. 2021 May 19;19(5):e3001009. doi: 10.1371/journal.pbio.3001009 (PMC8168858; doi:10.1371/journal.pbio.3001009)
Supplement: S2 Table — Continuous predictors were Z-transformed prior to model fitting. Bold italicized estimates indicate that the 95% credible intervals do not span zero. lnCV, log coefficient of variation; MLMR, multilevel meta-regression. (DOCX) [file pbio.3001009.s009.docx]

**S2 Table.** Conditional estimates and 95% credible intervals for lnCV, obtained from multi-level regression (MLMR) models of control group infarct volume. Continuous predictors were Z-transformed prior to model fitting. Bold italicized estimates indicate that the 95% credible intervals do not span zero.

| **Parameter** | $lnCV (\beta)$ | **LCI** | **UCI** |
| --- | --- | --- | --- |
| ***Intercept*** | ***-2.262*** | ***-3.024*** | ***-1.501*** |
| Sex _FEMALE_ | 0.328 | -0.112 | 0.768 |
| Sex _MALE_ | 0.307 | -0.075 | 0.689 |
| InductionMethod _EMBOLIC_ | 0.506 | -0.169 | 1.182 |
| InductionMethod _ENDOTHELIN_ | 0.500 | -0.166 | 1.167 |
| InductionMethod _FILAMENTAL_ | 0.086 | -0.537 | 0.709 |
| InductionMethod _DIRECT/MECHANICAL_ | 0.140 | -0.490 | 0.770 |
| InductionMethod _PHOTOTHROMBOSIS_ | 0.297 | -0.393 | 0.986 |
| ***InductionMethod _SPONTANEOUS_*** | ***1.118*** | ***0.191*** | ***2.045*** |
| ***IschaemiaModel _TEMPORARY_*** | ***0.206*** | ***0.087*** | ***0.326*** |
| IschaemiaModel _THROMBOTIC_ | 0.050 | -0.240 | 0.341 |
| Anesthesia _BARBITURATES_ | 0.050 | -0.147 | 0.248 |
| Anesthesia _INHALATION_ | 0.041 | -0.140 | 0.223 |
| ***TemperatureControl _YES_*** | ***0.181*** | ***0.041*** | ***0.321*** |
| PhysiologyMonitored _YES_ | 0.034 | -0.070 | 0.138 |
| ***AssessTime*** | ***0.053*** | ***0.012*** | ***0.095*** |
| ***MidWeight*** | ***0.092*** | ***0.045*** | ***0.139*** |
