# Supplementary material for: Meta-analysis of variation suggests that embracing variability improves both replicability and generalizability in preclinical research
Source: PLoS Biol. 2021 May 19;19(5):e3001009. doi: 10.1371/journal.pbio.3001009 (PMC8168858; doi:10.1371/journal.pbio.3001009)
Supplement: S3 Table — Treatment effects (DrugGroup) are ordered from groups that produce, on average, the greatest reduction in infarct volume (i.e., the most effective, as indicated by most negative estimates of lnRR) to groups that are, on average, the least effective. lnCVR, log coefficient of variation ratio; lnRR, log response ratio; MLMR, multilevel meta-regression. (DOCX) [file pbio.3001009.s010.docx]

**S3 Table.** Unconditional (marginalized) estimates and 95% credible intervals for lnRR and lnCVR, obtained from multi-level regression (MLMR) models of infarct volume in treatment/control groups. Treatment effects (DrugGroup) are ordered from groups that produce, on average, the greatest reduction in infarct volume (i.e. the most effective, as indicated by most negative estimates of lnRR) to groups that are, on average, the least effective.

| Parameter | lnRR | | | lnCVR | | |
| --- | --- | --- | --- | --- | --- | --- |
|  | $\beta$ | LCI | UCI | $\beta$ | LCI | UCI |
| Sex _MALE_ | -0.417 | -0.478 | -0.356 | 0.300 | 0.223 | 0.378 |
| Sex _FEMALE_ | -0.358 | -0.497 | -0.218 | 0.136 | -0.068 | 0.341 |
| Sex _BOTH_ | -0.233 | -0.451 | -0.015 | -0.017 | -0.323 | 0.288 |
| DrugGroup _HYPOTHERMIA_ | -0.687 | -0.775 | -0.599 | 0.470 | 0.349 | 0.591 |
| DrugGroup _OMEGA-3_ | -0.631 | -0.770 | -0.491 | 0.402 | 0.208 | 0.595 |
| DrugGroup _GTPase INHIBITOR_ | -0.546 | -0.737 | -0.355 | 0.393 | 0.122 | 0.665 |
| DrugGroup _ESTROGEN_ | -0.535 | -0.702 | -0.368 | 0.349 | 0.111 | 0.587 |
| DrugGroup _PPAR-GAMMA AGONIST_ | -0.531 | -0.652 | -0.410 | 0.295 | 0.115 | 0.475 |
| DrugGroup _NOOTROPIC_ | -0.524 | -0.869 | -0.179 | 0.277 | -0.201 | 0.754 |
| DrugGroup _ANTI-INFLAMMATORY_ | -0.502 | -0.683 | -0.322 | 0.334 | 0.084 | 0.584 |
| DrugGroup _IMMUNOSUPPRESSANT_ | -0.477 | -0.619 | -0.334 | 0.448 | 0.248 | 0.649 |
| DrugGroup _ANTIOXIDANT_ | -0.457 | -0.571 | -0.343 | 0.339 | 0.177 | 0.501 |
| DrugGroup _VITAMIN_ | -0.422 | -0.620 | -0.224 | 0.329 | 0.038 | 0.620 |
| DrugGroup _MK801_ | -0.399 | -0.540 | -0.258 | 0.377 | 0.169 | 0.585 |
| DrugGroup _GROWTH FACTOR_ | -0.360 | -0.464 | -0.256 | 0.291 | 0.143 | 0.439 |
| DrugGroup _HMG-CoA REDUCTASE ANTAGONIST_ | -0.360 | -0.497 | -0.222 | 0.331 | 0.134 | 0.529 |
| DrugGroup _THROMBOLYTICS_ | -0.351 | -0.446 | -0.256 | 0.160 | 0.031 | 0.289 |
| DrugGroup _CITOCHOLINE_ | -0.340 | -0.520 | -0.161 | -0.058 | -0.332 | 0.215 |
| DrugGroup _ANTIBIOTIC_ | -0.331 | -0.632 | -0.031 | 0.220 | -0.178 | 0.619 |
| DrugGroup _EXERCISE_ | -0.322 | -0.466 | -0.179 | 0.193 | -0.008 | 0.393 |
| DrugGroup _NOS INHIBITOR_ | -0.306 | -0.411 | -0.202 | 0.189 | 0.041 | 0.337 |
| DrugGroup _ANTIDEPRESSANT_ | -0.285 | -0.451 | -0.118 | 0.344 | 0.104 | 0.584 |
| DrugGroup _NO DONOR_ | -0.257 | -0.420 | -0.093 | 0.318 | 0.071 | 0.565 |
| DrugGroup _STEM CELLS_ | -0.222 | -0.311 | -0.132 | 0.196 | 0.068 | 0.323 |
| DrugGroup _ANGIOTENSIN RECEPTOR BLOCKER (ARB)_ | -0.110 | -0.642 | 0.423 | 0.306 | -0.575 | 1.187 |
| DrugGroup _HBOT_ | -0.098 | -0.634 | 0.438 | -0.603 | -1.483 | 0.277 |
| DrugGroup _MIXED TRAINING_ | -0.009 | -0.259 | 0.241 | -0.140 | -0.513 | 0.234 |
| DrugGroup _TRAINING_ | 0.048 | -0.242 | 0.338 | -0.007 | -0.399 | 0.386 |
| DrugGroup _ENVIRONMENT_ | 0.071 | -0.166 | 0.308 | -0.077 | -0.420 | 0.265 |
