# Supplementary material for: Meta-analysis of variation suggests that embracing variability improves both replicability and generalizability in preclinical research
Source: PLoS Biol. 2021 May 19;19(5):e3001009. doi: 10.1371/journal.pbio.3001009 (PMC8168858; doi:10.1371/journal.pbio.3001009)
Supplement: S4 Table — Bold italicized estimates indicate that the 95% credible intervals do not span zero. lnCVR, log coefficient of variation ratio; lnRR, log response ratio; MLMR, multilevel meta-regression. (DOCX) [file pbio.3001009.s011.docx]

**S4 Table.** Conditional estimates and 95% credible intervals for lnRR and lnCVR, obtained from multi-level regression (MLMR) models of infarct volume in treatment/control groups. Bold italicized estimates indicate that the 95% credible intervals do not span zero.

| **Parameter** | **lnRR** | | | **lnCVR** | | |
| --- | --- | --- | --- | --- | --- | --- |
|  | $\beta$ | LCI | UCI | $\beta$ | LCI | UCI |
| Intercept | ***-0.695*** | ***-0.783*** | ***-0.607*** | ***0.488*** | ***0.367*** | ***0.609*** |
| Sex _BOTH_ | 0.184 | -0.031 | 0.399 | ***-0.318*** | ***-0.623*** | ***-0.013*** |
| Sex _FEMALE_ | 0.059 | -0.079 | 0.197 | -0.164 | -0.369 | 0.042 |
| DrugGroup _ANGIOTENSIN RECEPTOR BLOCKER (ARB)_ | ***0.577*** | ***0.043*** | ***1.111*** | -0.164 | -1.048 | 0.720 |
| DrugGroup _ANTI-INFLAMMATORY_ | 0.185 | -0.002 | 0.371 | -0.136 | -0.398 | 0.127 |
| DrugGroup _ANTIBIOTIC_ | ***0.355*** | ***0.052*** | ***0.658*** | -0.250 | -0.654 | 0.155 |
| DrugGroup _ANTIDEPRESSANT_ | ***0.402*** | ***0.230*** | ***0.574*** | -0.126 | -0.378 | 0.126 |
| DrugGroup _ANTIOXIDANT_ | ***0.229*** | ***0.107*** | ***0.351*** | -0.131 | -0.311 | 0.048 |
| DrugGroup _CITOCHOLINE_ | ***0.346*** | ***0.160*** | ***0.533*** | ***-0.528*** | ***-0.813*** | ***-0.244*** |
| DrugGroup _ENVIRONMENT_ | ***0.758*** | ***0.517*** | ***0.998*** | ***-0.547*** | ***-0.899*** | ***-0.196*** |
| DrugGroup _ESTROGEN_ | 0.152 | -0.023 | 0.326 | -0.121 | -0.375 | 0.133 |
| DrugGroup _EXERCISE_ | ***0.365*** | ***0.215*** | ***0.514*** | ***-0.277*** | ***-0.491*** | ***-0.063*** |
| DrugGroup _GROWTH FACTOR_ | ***0.327*** | ***0.214*** | ***0.439*** | ***-0.179*** | ***-0.345*** | ***-0.013*** |
| DrugGroup _HBOT_ | ***0.589*** | ***0.050*** | ***1.128*** | ***-1.073*** | ***-1.958*** | ***-0.188*** |
| DrugGroup _HMG-CoA REDUCTASE ANTAGONIST_ | ***0.327*** | ***0.183*** | ***0.471*** | -0.139 | -0.350 | 0.073 |
| DrugGroup _IMMUNOSUPPRESSANT_ | ***0.210*** | ***0.062*** | ***0.358*** | -0.022 | -0.236 | 0.192 |
| DrugGroup _MIXED_ _TRAINING_ | ***0.678*** | ***0.420*** | ***0.935*** | ***-0.610*** | ***-0.995*** | ***-0.224*** |
| DrugGroup _MK801_ | ***0.288*** | ***0.140*** | ***0.435*** | -0.093 | -0.315 | 0.129 |
| DrugGroup _NOOTROPIC_ | 0.163 | -0.185 | 0.511 | -0.193 | -0.677 | 0.291 |
| DrugGroup _NO DONOR_ | ***0.430*** | ***0.260*** | ***0.600*** | -0.152 | -0.411 | 0.108 |
| DrugGroup _NOS INHIBITOR_ | ***0.381*** | ***0.265*** | ***0.496*** | ***-0.281*** | ***-0.450*** | ***-0.111*** |
| DrugGroup _OMEGA-3_ | 0.056 | -0.088 | 0.201 | -0.068 | -0.275 | 0.138 |
| DrugGroup _PPAR-GAMMA AGONIST_ | ***0.156*** | ***0.026*** | ***0.285*** | -0.175 | -0.371 | 0.022 |
| DrugGroup _GTPase INHIBITOR_ | 0.140 | -0.055 | 0.336 | -0.077 | -0.358 | 0.205 |
| DrugGroup _STEM CELLS_ | ***0.465*** | ***0.366*** | ***0.564*** | ***-0.274*** | ***-0.424*** | ***-0.125*** |
| DrugGroup _THROMBOLYTICS_ | ***0.336*** | ***0.233*** | ***0.438*** | ***-0.310*** | ***-0.458*** | ***-0.162*** |
| DrugGroup _TRAINING_ | ***0.735*** | ***0.442*** | ***1.028*** | ***-0.477*** | ***-0.877*** | ***-0.077*** |
| DrugGroup _VITAMIN_ | ***0.265*** | ***0.061*** | ***0.468*** | -0.141 | -0.443 | 0.162 |
