# Supplementary material for: Meta-analysis of variation suggests that embracing variability improves both replicability and generalizability in preclinical research
Source: PLoS Biol. 2021 May 19;19(5):e3001009. doi: 10.1371/journal.pbio.3001009 (PMC8168858; doi:10.1371/journal.pbio.3001009)
Supplement: S6 Table — The intercept here represents studies in which only “Female” sex was used. Bold italicized estimates indicate that the 95% credible intervals do not span zero. lnCVR, log coefficient of variation ratio; lnRR, log response ratio; MLMR, multilevel meta-regression. (DOCX) [file pbio.3001009.s013.docx]

**S6 Table.** Conditional estimates and 95% credible intervals for lnRR and lnCVR, obtained from contrast multi-level regression (MLMR) models to assess the effect of sex on infarct volume. The intercept here represents studies in which only “Female” sex was used. Bold italicized estimates indicate that the 95% credible intervals do not span zero.

| Parameters | lnRR | | | lnCVR | | |
| --- | --- | --- | --- | --- | --- | --- |
|  | $\beta$ | LCI | UCI | $\beta$ | LCI | UCI |
| Intercept | **-0.636** | **-0.795** | **-0.476** | **0.324** | **0.091** | **0.558** |
| Sex _BOTH_ | 0.125 | -0.103 | 0.352 | -0.154 | -0.482 | 0.174 |
| Sex _MALE_ | -0.059 | -0.197 | 0.079 | 0.164 | -0.042 | 0.369 |
| DrugGroup _ANGIOTENSIN RECEPTOR BLOCKER (ARB)_ | ***0.577*** | ***0.043*** | ***1.111*** | -0.164 | -1.048 | 0.720 |
| DrugGroup _ANTI-INFLAMMATORY_ | 0.185 | -0.002 | 0.371 | -0.136 | -0.398 | 0.127 |
| DrugGroup _ANTIBIOTIC_ | ***0.355*** | ***0.052*** | ***0.658*** | -0.250 | -0.654 | 0.155 |
| DrugGroup _ANTIDEPRESSANT_ | ***0.402*** | ***0.230*** | ***0.574*** | -0.126 | -0.378 | 0.126 |
| DrugGroup _ANTIOXIDANT_ | ***0.229*** | ***0.107*** | ***0.351*** | -0.131 | -0.311 | 0.048 |
| DrugGroup _CITOCHOLINE_ | ***0.346*** | ***0.160*** | ***0.533*** | ***-0.528*** | ***-0.813*** | ***-0.244*** |
| DrugGroup _ENVIRONMENT_ | ***0.758*** | ***0.517*** | ***0.998*** | ***-0.547*** | ***-0.899*** | ***-0.196*** |
| DrugGroup _ESTROGEN_ | 0.152 | -0.023 | 0.326 | -0.121 | -0.375 | 0.133 |
| DrugGroup _EXERCISE_ | ***0.365*** | ***0.215*** | ***0.514*** | ***-0.277*** | ***-0.491*** | ***-0.063*** |
| DrugGroup _GROWTH FACTOR_ | ***0.327*** | ***0.214*** | ***0.439*** | ***-0.179*** | ***-0.345*** | ***-0.013*** |
| DrugGroup _HBOT_ | ***0.589*** | ***0.050*** | ***1.128*** | ***-1.073*** | ***-1.958*** | ***-0.188*** |
| DrugGroup _HMG-CoA REDUCTASE ANTAGONIST_ | ***0.327*** | ***0.183*** | ***0.471*** | -0.139 | -0.350 | 0.073 |
| DrugGroup _IMMUNOSUPPRESSANT_ | ***0.210*** | ***0.062*** | ***0.358*** | -0.022 | -0.236 | 0.192 |
| DrugGroup _MIXED_ _TRAINING_ | ***0.678*** | ***0.420*** | ***0.935*** | ***-0.610*** | ***-0.995*** | ***-0.224*** |
| DrugGroup _MK801_ | ***0.288*** | ***0.140*** | ***0.435*** | -0.093 | -0.315 | 0.129 |
| DrugGroup _NOOTROPIC_ | 0.163 | -0.185 | 0.511 | -0.193 | -0.677 | 0.291 |
| DrugGroup _NO DONOR_ | ***0.430*** | ***0.260*** | ***0.600*** | -0.152 | -0.411 | 0.108 |
| DrugGroup _NOS INHIBITOR_ | ***0.381*** | ***0.265*** | ***0.496*** | ***-0.281*** | ***-0.450*** | ***-0.111*** |
| DrugGroup _OMEGA-3_ | 0.056 | -0.088 | 0.201 | -0.068 | -0.275 | 0.138 |
| DrugGroup _PPAR-GAMMA AGONIST_ | ***0.156*** | ***0.026*** | ***0.285*** | -0.175 | -0.371 | 0.022 |
| DrugGroup _GTPase INHIBITOR_ | 0.140 | -0.055 | 0.336 | -0.077 | -0.358 | 0.205 |
| DrugGroup _STEM CELLS_ | ***0.465*** | ***0.366*** | ***0.564*** | ***-0.274*** | ***-0.424*** | ***-0.125*** |
| DrugGroup _THROMBOLYTICS_ | ***0.336*** | ***0.233*** | ***0.438*** | ***-0.310*** | ***-0.458*** | ***-0.162*** |
| DrugGroup _TRAINING_ | ***0.735*** | ***0.442*** | ***1.028*** | ***-0.477*** | ***-0.877*** | ***-0.077*** |
| DrugGroup _VITAMIN_ | ***0.265*** | ***0.061*** | ***0.468*** | -0.141 | -0.443 | 0.162 |
