# Supplementary material for: Meta-analysis of variation suggests that embracing variability improves both replicability and generalizability in preclinical research
Source: PLoS Biol. 2021 May 19;19(5):e3001009. doi: 10.1371/journal.pbio.3001009 (PMC8168858; doi:10.1371/journal.pbio.3001009)
Supplement: S7 Table — For our second-order meta-regression, we first separated our rat infarct volume data by occlusion methods. For each occlusion method data, we conducted a MLMR to estimate heterogeneity (I2) in lnRR including our original random (study ID, effect size ID, and strain) and fixed effects (sex + drug treatment group). From our MLMR models, we extracted total I2 of lnRR and from this calculated the heterogeneity statistic lnH. lnH is a preferable effect size for downstream analyses as it is unbounded and has a relatively well-defined standard error to act as a measure of its precision [61 in main text]. Using the square of the standard error of lnH as the sampling variance and lnH as our response variable, we then fit a second-order meta-regression using the lnCV estimates of each occlusion method as a fixed predictor and effect size ID as a random effect (σ2Residual = 0.200). Unconditional estimates of lnCV were obtained from our MLMR models of methodological variability (S1 Table) described in our main text. Estimates and 95% credible intervals from this second-order MLMR model is reported below. Estimates with credible intervals that do not span zero are considered statistically significant. See S3 Fig for a line plot depicting the relationship between lnH and lnCV with the model fitted line. lnCV, log coefficient of variation; lnRR, log response ratio; MLMR, multilevel meta-regression. (DOCX) [file pbio.3001009.s014.docx]

**S7 Table.** Consistency in drug treatment outcomes across variability induced by occlusion methodologies. For our second-order meta-regression, we first separated our rat infarct volume data by occlusion methods. For each occlusion method data, we conducted a MLMR to estimate heterogeneity (*I*^2^) in lnRR including our original random (study ID, effect size ID and strain) and fixed effects (sex + drug treatment group). From our MLMR models, we extracted total *I*^2^ of lnRR and from this calculated the heterogeneity statistic ln*H*. ln*H* is a preferable effect size for downstream analyses as it is unbounded and has a relatively well-defined standard error to act as a measure of its precision [61 in main text]. Using the square of the standard error of ln*H* as the sampling variance and ln*H* as our response variable, we then fit a second-order meta-regression using the lnCV estimates of each occlusion method as a fixed predictor, and effect size ID as a random effect (${\sigma^{2}}_{Residual}$ = 0.200). Unconditional estimates of lnCV were obtained from our MLMR models of methodological variability (Table S1) described in our main text. Estimates and 95% credible intervals from this second-order MLMR model is reported below. Estimates with credible intervals that do not span zero are considered statistically significant. See S3 Fig for a line-plot depicting the relationship between ln*H* and lnCV with the model fitted line.

| Parameters | $lnH (\beta)$ | LCI | UCI |
| --- | --- | --- | --- |
| Intercept | -0.266 | -1.956 | 1.424 |
| lnCV | -0.876 | -2.047 | 0.295 |
