# Supplementary material for: Meta-analysis of variation suggests that embracing variability improves both replicability and generalizability in preclinical research
Source: PLoS Biol. 2021 May 19;19(5):e3001009. doi: 10.1371/journal.pbio.3001009 (PMC8168858; doi:10.1371/journal.pbio.3001009)
Supplement: S8 Table — Effect sizes and sampling variances used in meta-analysis of variance (a) across methodological predictors and (b) across drug treatment groups. Equations and the model type in which the effect size was used are also given. x and s are the mean and SD of the group infarct volume, n is the sample size, CV is the coefficient of variation, and ρ is the correlation between the mean and standard deviation on the log scale (ρ is assumed to be 0*). Subscripts C and E refer to control and treatment groups, respectively. (DOCX) [file pbio.3001009.s015.docx]

**S8 Table.** Effect sizes and sampling variances used in meta-analysis of variance (a) across methodological predictors and (b) across drug treatment groups. Equations and the model type in which the effect size was used are also given. $\underline{x}$ and $s$ is the mean and SD of the group infarct volume, $n$ is the sample size, CV is the coefficient of variation, and $\rho$ is the correlation between the mean and standard deviation on the log scale ($\rho$ is assumed to be 0*). Subscripts C and E refer to control and treatment groups, respectively.

| Effect Size | Outcome Measure | Equation | Sampling Variance (*s*^2^ _Effect Size_) | Model |
| --- | --- | --- | --- | --- |
| 1. **Meta-analysis of methodological predictors** | | | | |
| $lnCV$ | Coefficient of Variation | $ln\left( \frac{s}{\underline{x}} \right)+ \frac{1}{2(n-1)}$ | $\frac{s^{2}}{n\underline{x}^{2}}+ \frac{1}{2(n-1)}- 2\rho\sqrt{\frac{s^{2}}{n\underline{x}^{2}}\frac{1}{2(n_{C}-1)}}$ | Arm-based / Meta-regression |
| 1. **Meta-analysis of drug treatment** | | | | |
| $lnRR$ | Mean | $ln\left( \frac{\underline{x}_{E}}{\underline{x}_{C}} \right)$ | $\frac{s_{C}^{2}}{n_{C}\underline{x}_{C}^{2}}+ \frac{s_{E}^{2}}{n_{E}\underline{x}_{E}^{2}}$ | Contrast-based |
| $lnCVR$ | Coefficient of Variance | $ln\left( \frac{{CV}_{E}}{{CV}_{C}} \right)+ \frac{1}{2(n_{E}-1)}- \frac{1}{2(n_{C}-1)}$ | $\frac{s_{C}^{2}}{n_{C}\underline{x}_{C}^{2}}+\frac{1}{2(n_{C}-1)}-2\rho\sqrt{\frac{s_{C}^{2}}{n_{C}\underline{x}_{C}^{2}}\frac{1}{2(n_{C}-1)}}$ +  $\frac{s_{E}^{2}}{n_{E}\underline{x}_{E}^{2}}+\frac{1}{2(n_{E}-1)}-2\rho\sqrt{\frac{s_{E}^{2}}{n_{E}\underline{x}_{E}^{2}}\frac{1}{2\left( n_{E}-1 \right)}}$ | Contrast-based |

*Senior AM, Viechtbauer W, Nakagawa S. Revisiting and expanding the meta‐analysis of variation: The log coefficient of variation ratio. Res Synth Methods. 2020; 553–567.
