# Supplementary material for: Meta-analysis of variation suggests that embracing variability improves both replicability and generalizability in preclinical research
Source: PLoS Biol. 2021 May 19;19(5):e3001009. doi: 10.1371/journal.pbio.3001009 (PMC8168858; doi:10.1371/journal.pbio.3001009)
Supplement: S10 Table — Continuous predictors were Z-transformed prior to model fitting. lnCV, log coefficient of variation; MLMR, multilevel meta-regression. (DOCX) [file pbio.3001009.s017.docx]

**S10 Table.** Sensitivity model estimates (unconditional) and 95% credible intervals for lnCV, obtained from multi-level regression (MLMR) models of control group infarct volume. Continuous predictors were Z-transformed prior to model fitting.

| Fixed Parameters | $lnCV (\beta)$ | LCI | UCI |
| --- | --- | --- | --- |
| Sex _BOTH_ | -1.757 | -2.164 | -1.350 |
| Sex _FEMALE_ | -1.429 | -1.679 | -1.179 |
| Sex _MALE_ | -1.456 | -1.587 | -1.325 |
| InductionMethod _COLLAGENASE_ | -1.634 | -2.272 | -0.996 |
| InductionMethod _EMBOLIC_ | -1.104 | -1.365 | -0.843 |
| InductionMethod _ENDOTHELIN_ | -1.264 | -2.026 | -0.502 |
| InductionMethod _FILAMENTAL_ | -1.729 | -2.224 | -1.234 |
| InductionMethod _DIRECT/MECHANICAL_ | -1.641 | -2.276 | -1.005 |
| InductionMethod _PHOTOTHROMBOSIS_ | -1.471 | -2.260 | -0.682 |
| InductionMethod _SPONTANEOUS_ | -0.529 | -1.526 | 0.467 |
| IschaemiaModel _PERMANENT_ | -1.583 | -1.735 | -1.431 |
| IschaemiaModel _TEMPORARY_ | -1.385 | -1.525 | -1.245 |
| IschaemiaModel _THROMBOTIC_ | -1.544 | -1.819 | -1.269 |
| Anesthesia _KETAMINE_ | -1.522 | -1.721 | -1.323 |
| Anesthesia _INHALATION_ | -1.454 | -1.590 | -1.318 |
| Anesthesia _BARBITURATES_ | -1.457 | -1.622 | -1.293 |
| TemperatureControl _NO_ | -1.590 | -1.767 | -1.414 |
| TemperatureControl _YES_ | -1.434 | -1.567 | -1.301 |
| PhysiologyMonitored _NO_ | -1.484 | -1.624 | -1.345 |
| PhysiologyMonitored _YES_ | -1.443 | -1.584 | -1.301 |
| AssessTime | -1.408 | -1.545 | -1.272 |
| MidWeight | -1.419 | -1.557 | -1.281 |
