# Supplementary material for: Meta-analysis of variation suggests that embracing variability improves both replicability and generalizability in preclinical research
Source: PLoS Biol. 2021 May 19;19(5):e3001009. doi: 10.1371/journal.pbio.3001009 (PMC8168858; doi:10.1371/journal.pbio.3001009)
Supplement: S12 Table — Estimates (%) are shown for MLMAs and MLMR models. lnCV, log coefficient of variation; lnCVR, log coefficient of variation ratio; lnRR, log response ratio; MLMA, multilevel meta-analysis; MLMR, multilevel meta-regression. (DOCX) [file pbio.3001009.s019.docx]

**S12 Table.** Sensitivity model estimates of heterogeneity ($I^{2}$) for analyses of methodology on variability (lnCV) and drug treatment on mean (lnRR) and variance (lnCVR) in rat infarct volume. Estimates (%) are shown for multi-level meta-analyses (MLMA) and multilevel meta-regression (MLMR) models.

| Model | Total | Study | Strain | Publication quality | Residual  (within-study) |
| --- | --- | --- | --- | --- | --- |
| *lnCV* |  |  |  |  |  |
| MLMA | 93.8% | 48.3% | 1.5% | 1.5% | 42.5% |
| MLMR | 93.4% | 46.4% | 2.1% | 0.3% | 44.7% |
| *lnRR* |  |  |  |  |  |
| MLMA | 95.7% | 54.2% | 1.6% | 0.7% | 39.2% |
| MLMR | 94.9% | 46.1% | 2.0% | 0.8% | 46.1% |
| *lnCVR* |  |  |  |  |  |
| MLMA | 71.3% | 38.7% | 0.8% | 0.3% | 31.4% |
| MLMR | 97.3% | 50.0% | 1.3% | 0.3% | 45.7% |
