# Supplementary material for: Meta-analysis of variation suggests that embracing variability improves both replicability and generalizability in preclinical research
Source: PLoS Biol. 2021 May 19;19(5):e3001009. doi: 10.1371/journal.pbio.3001009 (PMC8168858; doi:10.1371/journal.pbio.3001009)
Supplement: S13 Table — This procedure fits the square root of sampling variance as a moderator (slope estimate and 95% credible intervals shown in the first half of the table). If this estimate is significant, we then fit the sampling variance (second half of the table). The intercept from this latter model indicates a “potentially” bias-corrected, modified meta-analytic mean. In our case, the biased-corrected estimate is a 28.0% decline, compared with the original estimate without correction, which is a 29.6% decline. These values indicate that although this analysis detected a sign of publication bias, the effect of this bias is very small (1.6% difference). Bold italicized estimates indicate that the 95% credible intervals do not span zero. lnRR, log response ratio; PEESE, precision-effect estimate with standard errors; PET, precision-effect test. (DOCX) [file pbio.3001009.s020.docx]

**S13 Table.** Results from Egger regression (PET-PEESE) on lnRR to test for publication bias. This procedure fits the square-root of sampling variance as a moderator (slope estimate and 95% credible intervals shown in the first half of the table). If this estimate is significant, we then fit the sampling variance (second half of the table). The intercept from this latter model indicates a ‘potentially’ bias-corrected, modified meta-analytic mean. In our case, the biased-corrected estimate is a 28.0% decline, compared with the original estimate without correction, which is a 29.6% decline. These values indicate that although this analysis detected a sign of publication bias, the effect of this bias is very small (1.6% difference). Bold italicized estimates indicate that the 95% credible intervals do not span zero.

| **Parameter** | **lnRR**$(\beta)$ | **LCI** | **UCI** |
| --- | --- | --- | --- |
| ***Intercept*** | ***-0.1525*** | ***-0.2102*** | ***-0.0949*** |
| **sqrt(sampling variance)** | ***-1.5185*** | ***-1.7131*** | ***-1.3239*** |
| ***Intercept*** | ***-0.3285*** | ***-0.3813*** | ***-0.2757*** |
| ***sampling variance*** | ***-1.8964*** | ***-2.2141*** | ***-1.5787*** |
