# Supplementary material for: Meta-analysis of variation suggests that embracing variability improves both replicability and generalizability in preclinical research
Source: PLoS Biol. 2021 May 19;19(5):e3001009. doi: 10.1371/journal.pbio.3001009 (PMC8168858; doi:10.1371/journal.pbio.3001009)
Supplement: Attachment — Submitted filename: Response to Reviewers.pdf [file pbio.3001009.s021.pdf]

PBIOLOGY-D-20-03180R1
Title: Embrace heterogeneity to improve reproducibility: A perspective from meta-analysis of variation in preclinical research

Dear Professor Roland G Roberts,

We have substantially revised this ms and addressed all of the insightful comments from yourself and the two reviewers. The major changes to the ms are that: (i) we now provide additional analyses to support our central claim that using methodologies that induce variability in disease-states lead to more generalizable outcomes; (ii) we now have restructured and rewritten sections of the manuscript to provide clearer recommendations that arise from this manuscript; and (iii) we have provided further sensitivity analyses that take into account concerns regarding potential within-study biases and the use of our meta-analytic effect size lnCVR. None of our major insights or arguments have changed in this revision, but we hope that the reviewers are now more convinced as to the robustness and significance of the perspectives that we present.

Below we describe our response to each of the reviewers comments. Line numbers match the revised manuscript with changes highlighted. We hope that you find that this revised manuscript is much improved.

Yours sincerely,

Takuji Usui

<u>Senior Editor: Roland G Roberts</u>
You'll see that although there's some positivity from both reviewers, each of them suggests some additional analyses, and reviewer #2 suggests that without such analyses the central claims are poorly supported (and without those claims this article is of specialist interest only). There are also questions about how the lessons learned would be applied in real life. In light of the reviews (below), we will not be able to accept the current version of the manuscript, but we would welcome re-submission of a much-revised version that takes into account the reviewers' comments. We cannot make any decision about publication until we have seen the revised manuscript and your response to the reviewers' comments. Your revised manuscript is also likely to be sent for further evaluation by the reviewers.

*** We thank Prof Roberts for the opportunity to resubmit, and gratefully appreciate the thoughtful feedback and comments provided by yourself and the reviewers. One of our central arguments was to advocate for the diversification of disease-states to improve generalizability, by using methodologies that induce more variation (as quantified through our meta-analysis of control groups) rather than minimizing variability through standardization. At the suggestion of reviewer #2, we now provide a formal second-order meta-regression that empirically quantifies the relationship between variability in baseline, disease-states induced by methodology (occlusion methods) and heterogeneity in drug treatment outcomes. Consistent with our arguments, we find a negative relationship between lnCV (variability induced by occlusion methods) and ln*H* (heterogeneity in drug treatment outcomes), suggesting that methodologies that generate greater variability in disease-states

are associated with more consistent mean drug treatment outcomes. We note, however, that our slope estimate is statistically non-significant (slope = -0.876 -2.047 to 0.295) as our analysis could only be based on a small number of unbalanced data points. We now refer to this finding in the main manuscript (lines 371-385) and report the full analytical methods and results in the supplementary text (Fig S2 for line plot assessing relationship between lnCV and ln*H*; Table S7 for full model coefficients and details of analysis).

With regards to practical recommendations that arise from this manuscript as noted by reviewer #1, we now provide more explicit recommendations for how to include heterogeneity in experimental designs in our Discussion (lines 334-352). We hope that our new analyses and clarifications provided in the manuscript clarify the value and utility of our perspective provided by this meta-analysis.

__**Reviewer #1:**__

(1) A major limitation of this study is it depends on reported outcomes, which are prone to biases. The authors tested for signs of publication bias, which indicated there was a very small bias, however there are also within-study biases (e.g., selective reporting, lack of or unproper randomization, lack of blinding, etc). Although it might be hard to detect the impact these have on the estimates provided like publication bias, it is probably worth noting in the main text the limitation and/or the impacts of within-study biases on this analysis, as well as the results that there appears to be a very small publication bias (which is currently only mentioned in the supplement and methods).

*** We thank the reviewer for this comment and agree entirely that within-study biases could influence reported outcomes, both in our study and in meta-analyses more generally. We now provide sensitivity analyses to quantitatively assess the impact of within-study biases on our estimates of lnRR (as the reviewer points out, traditional publication bias tests are unlikely to detect this kind of within-study biases). In our analyses we included, as a random effect, a metric of publication quality ("high" or "low") developed according to guidelines set out by the Stroke Academic Industry Roundtable (STAIR; Macleod et al. 2004 *Stroke*, 35, 1203-1208). This metric scores studies based on whether they controlled for various within-study biases, including selection bias (i.e. lack of randomization) as well as performance and detection bias (i.e. lack of blinding of interventions and/or outcome measures). Our models show that the I^2 attributable to publication quality was minimal (0.7%), suggesting that publication quality accounted for very little in terms of differences in mean treatment outcomes. Our estimates of lnRR also changed very little, with no qualitative changes to the main results reported on drug treatment effects. We now provide the results of our sensitivity analysis for lnRR in our supplementary text (Figs S6 for forest plot, Table S11 in S1 Text for unconditional model estimates, and Table S12 for heterogeneity estimates). We also make reference to this point on within-study biases in the main manuscript (lines 583-593).

We also conducted this additional sensitivity analysis for lnCV and lnCVR. However, whilst within-study biases could clearly affect population mean estimates, we have no *apriori* hypothesis for what such biases would do to our estimates of variance. Nonetheless, for consistency we report sensitivity analyses of lnCV (Fig S5 for forest plot; Tables S10 for unconditional model estimates) and lnCVR (Fig S6 for forest plot; Table S11 for

unconditional estimates) in the supplementary text. Again, our model estimates of lnCV and lnCVR changed very little with this additional random effect, with no qualitative changes to the reported main results. Finally, we now also reference our publication bias results (Egger regression of lnRR) in our main manuscript (lines 593-603).

(2) I agree on the logical exploration of whether sex of the groups used in the experiments affected lnRR or lnCVR, however as Figure 1 illustrates the greatest source of variability was with the occlusion methods (spontaneous in particular). Related to this would be knowing the relationship of methodology and drug treatments (e.g., do occlusion methods of higher variability (figure 1) associate with drug groups that also have higher variability (figure 2?).

*** We thank the reviewers' suggestions here and below on linking methodological variability to consistency in drug treatment effects. We now provide a second-order meta-regression that quantifies the relationship between variability induced by occlusion methods (lnCV) and heterogeneity in drug treatment outcomes. We describe details of our analysis and results in our response below.

(3) Lines 272-274: Maybe by looking at how filament methods vs other methods impact efficacy (e.g., point above about fig 1 and 2) might strengthen this. This naturally would be subsetting the data in a manner where broad conclusions cannot be made, but using it as a case study (e.g., looking at how the use of the filamental approach vs 'non'-filamental approaches impacts the effect of drug treatment(s) on the difference in infarct volume) would help connect how variations in methodology lead to variations in drug treatment effects. This can also serve as a good example that might help the reader translate the findings from this study into their specific area of interest (related to point 4 below).

** To quantify the relationship between variability induced by different occlusion methodologies to consistency in drug treatment effects, we first subset the dataset into occlusion methods, and conducted a separate MLMR for each data subset to estimate heterogeneity (I^2) in lnRR (i.e. effect of drug). We included study, strain and effect size IDs as random terms in our model as before, and included our original fixed effects (sex + drug treatment group). From total I^2 of lnRR, we then calculated the heterogeneity statistic ln*H*, and its associated standard error estimates (Higgins & Thompson, 2002, British Medical Journal, 327, 557-560). We then fit a formal, second-order meta-regression including lnCV estimates of each occlusion method as a fixed predictor, effect size ID as a random effect, and the square of the standard error of ln*H* as the sampling variance.

For methods that generated greater variability (i.e. less negative lnCV estimates), we predicted greater consistency (lower ln*H*) in mean drug treatment outcomes. In agreement, our results show a negative relationship between lnCV and ln*H* (slope = -0.876 -2.047 to 0.295). While these results suggest the support of our underlying hypothesis, we note that this slope estimate is statistically non-significant and that our analysis could only be based on a small number of unbalanced data points. We refer to these results now in the main manuscript (lines 371-385) and report the full results and analytical methods in the Supplementary text (Fig S2; see Table S7 for full results and analysis details).

(4) I would recommend the authors expand their discussion/conclusions about how to incorporate heterogeneity into the design of experiments. The authors comment on the challenge in doing so (e.g., it is not ethically or practically possible to include all possible combinations), but leave the reader still having to navigate what the 'best practice' should be (or maybe more accurately how to utilize the information of variability in experimental design choices). That is, since new studies will always start highly constrained (e.g., specific animal model choices, specific methodological choices, etc), but need to incorporate more heterogeneity among the landscape of possibilities what considerations should a researcher consider outside selecting methodologies that generate variability. What would be undesirable is avoidance of high variability experimental designs, despite including more methodological heterogentiy, that are the most likely to be translatable. Related, it would be undesirable to include many experimental design choices early, exhausting resources and presenting ethical dilemmas, when only a few would be beneficial to purse earlier in the research process (and note: this is why sharing all outcomes are vital - not just positive findings). A paper the authors might consider related to this topic (e.g., the interplay between exploring the space of possible experimental combinations and how it impacts the constraints on reproducibility and generalizability):
https://journals.plos.org/plosbiology/article/comments?id=10.1371/journal.pbio.3000691

*** We thank the reviewer for this insight and recommendation. We completely agree that it would be undesirable to: 1) create an experiment that includes more heterogeneity in its design, but that does not translate to heterogeneity in outcomes; and 2) haphazardly incorporate heterogeneous designs when a few key variables could induce the amount of heterogenization required. We think that this is exactly the reason why it is necessary and useful to quantify how different methodological choices, from a spectrum of applied combinations, induce heterogeneity in outcomes through meta-analysis as done in our paper. Our best practice recommendation would then be to identify and only include methodological combinations that most effectively induce variability in our baseline control outcomes (from Fig. 1 and Table S1), and avoid using factors that only weakly induce variability.

In some cases, utilizing more heterogeneous designs may simply be achieved by replacing current designs with more variable methodologies (for example, through changing methods of inducing occlusion from one that creates less variability to one that creates more). In other cases, confounding factors that in traditional designs would be standardized (e.g. biological variation such as sex/strain, or other environmental effects), may need to be deliberately introduced into the design in a structured, formal approach (known as “controlled heterogenization”). This may be achieved through incorporating multiple levels of these categorical (e.g. strain) or continuous (e.g. time of assessment) confounds in a fully-crossed or randomized block design (Voekl et al. 2021 *Nature Methods*, 18, 3-7). Regardless of the approach taken to incorporate heterogeneity, it is vital that we know what methodologies induce the greatest amount of variability so that we can effectively utilize these methods from the vast spectrum of available combinations (for example, with increasing numbers of confounding variables, there is a rapid increase in the resulting number of blocks required for heterogenization). We now further emphasize the above points in our Discussion, and

include clarifications in our recommendation in our manuscript (lines 334-352). Overall, we argue that initially maximizing heterogeneity may be a more effective approach to finding boundary constraints of generalizability and reproducibility, as opposed to the current research paradigm that by design reduces the plausible space over which outcomes may be made generalizable and reproducible across studies.

Minor points:
Line 327 - Fig 3 not Fig 4

*** Thank you, this has been corrected.

The authors should include the number/percent of studies excluded (e.g., standard error was 0, small sample size, etc). A flow diagram (e.g., PRISMA) might be useful here.

*** We thank the reviewer for pointing this out. We now clarify the selection process in the main manuscript (lines 467 - 480) and also provide a PRISMA flow chart for the database query and selection process in a new supplementary figure (Fig S3).

Reviewer #2:

This is a well written and important paper that tries to make the case that limiting variability, while potentially increasing statistical power for an individual experiment, is detrimental when one considers the replicability of findings. The paper presents a meta-analysis of preclinical stroke studies, and examines how the variability of outcome depends on both the method for stroke induction and the treatment given. I agree with the authors arguments fully. The argument for the small sample sizes that are the norm in preclinical research is that the greatly reduced inter-individual variability drastically increases the power to detect experimental effects. This is true and may in some circumstance be justified (e.g. when one is trying to identify specific functions of individual circuits), however as soon as one begins to undertake research with clinical translatability there is a need for findings that generalise beyond tightly constrained boundaries.

*** We thank the reviewer for their kind comments.

While I agree with this point of view, the main issue I have is that I don't think the paper's analyses fully support these arguments. For example, the authors show that various methods of stroke induction differ in the variability of lesion volume induced but they do not provide any analyses supporting their argument that greater variability is of benefit. This is highlighted when in the discussion (lines 270-274) the authors highlight similar research that drew the opposite conclusion from the same data. I would hope that given the large number of studies available to the authors it might be possible to build on the current analyses to give some quantitative weight to the main arguments of the paper.

*** We thank the reviewer for their insightful comments on supporting analyses linking methodological variability and consistency in drug treatment effects. We now provide such an analysis and describe its relevance and caveats below.

For example, might it be possible to examine if stroke induction methods with a greater CV (e.g. embolic) show greater consistency in terms of treatment effects than methods with low CV (e.g. filamental). Could one perform a meta- regression: (mean CV stroke induction method CV) ~ I2 (for lnRR or a SMD), i.e. each data point would be a different stroke induction method (ideally a separate meta regression for each treatment type) ? If this isn't possible please do explain why and also discuss how one might explicitly test this. Without this kind of analysis the paper's title does not really represent the content and the paper would be better titled and presented as something of interest to preclinical stroke researchers rather than something of potentially broad relevance.

*** We greatly appreciate the reviewers' suggestions above on linking methodological variability to consistency in drug treatment effects. To this end, we now provide such an analysis, focusing on methods of stroke induction: (1) We first conducted a MLMR to estimate heterogeneity (I^2) in lnRR, separately for each type of stroke induction method. We included study, strain and effect size IDs as random terms in our model as before, and included our original fixed effects (sex + drug treatment group). (2) From total I^2 of lnRR, we then calculated the heterogeneity statistic ln*H*, which allows for estimation of standard error in our heterogeneity estimates (Higgins & Thompson, 2002, British Medical Journal, 327, 557-560). (3) Using the square of the standard error of ln*H* as the sampling variance and ln*H* as our response variable, we then fit a second-order meta-regression including lnCV estimates of each occlusion method as a fixed predictor, and effect size ID as a random effect.

For methods that generate greater variability in our base-line, control group outcomes (i.e. less negative lnCV estimates), we predict greater consistency in mean drug treatment outcomes (lower ln*H*). In agreement, our results show a negative relationship between lnCV and ln*H* (Fig S2; slope = -0.876 -2.047 to 0.295). We note, however, that our slope estimate is statistically non-significant, and our analysis could only be based on a small and unbalanced dataset. We now report these results in the main manuscript, with these caveats acknowledged (lines 371-385), and report the full results and analysis methods in the Supplementary text (Fig S2; Table S7 documents full results and analysis details).

A few other points:

1. Particularly with clinically relevant behavioural outcomes many outcome measures are not ratio scaled. This can be the case where the outcome measure does not have a zero limit or even with data that does have a zero limit, the mean-sd relationship often become nonlinear toward zero. This has led to erroneous conclusions when using the CVR (see the recent retraction of the Maslej et al 2020 in JAMA Psych). The current data looks like the assumption may be met but checking to see that the intercept is not significantly different from 0 for the SD~mean regression would be a good double check)

*** We thank the reviewer for this point of concern. As noted by the reviewer, we agree that this dataset should be less prone to violating assumptions of linearity in mean-sd relationships (our dataset does not concern behavioural outcomes but are of infarct size only). We previously presented this relationship on the natural scale, however, we now clarify that the linear relationship between mean and SD should be present on the logarithmic scale. Our log-log plot of mean-SD shows a linear relationship in both our control and treatment datasets (Figure S1). For clarity, we now present the slope estimates, associated 95% CIs, and a 1:1 line for visual reference.

As noted by the reviewer, CVR (and CV) assumes a linear slope coefficient of 1 between log(mean) and log(SD). In the Maslej et al. 2020 JAMA Psych paper, slope estimates were 0.22 and 0.25 for antidepressants and placebos, respectively, which the critics correctly argued were much closer to 0 and thus CVR was not an appropriate measure. Our slope estimates from a simple regression on the log scale are 0.758 (0.728 to 0.788) and 0.822 (0.791 to 0.854) for treatment and control groups, respectively (now reported on the S1 Figure legend). As the slope coefficient is much closer to 1 than 0, we argue that CVR is an appropriate measure of variance in our dataset. The commentary and follow-up analysis to the Maslej et al. 2020 paper also noted that since reported outcomes are on different (depression) scales, regressing over all of these terms can lead to biased slope estimates, showing that the slope coefficient was actually ~0.1 with a varying intercept model that accounted for scale (Volkmann et al. 2020 PLoS ONE, 15: e0241497). We therefore additionally fit a linear mixed-effects model of log(mean) and log(SD) with a random intercept of “Unit” that describes the unit measurement of infarct volume. Slope estimates from this model are still considerably closer to 1 than 0 in both our treatment (0.769, 0.740 to 0.799) and control (0.824, 0.793 to 0.855) datasets. Lastly, as described below, we have also now ran an arm-based meta-analysis of lnSD fitting Z-transformed log mean infarct size as a predictor.

2. Related to the above point, the increase in CV for most of the treatments is I expect driven by reduction in mean volume for these treatments, as implied by figure 4 in which there it is clear that studies with the more effective treatment show the greater lnCVR. If there is not an almost perfectly linear relationship between SD and mean, then I worry that the same issue that affected the recently retracted Maslej et al paper may be in play here and wonder if the hierarchical model with ln SD as the response might be preferable (the initial analysis using endpoint treatment scores suggested greater CV with treatment due to the fact that treated groups have lower endpoint scores, a reanalysis of this data using the hierarchical model with ln SD as the response subsequently showed that variability did not differ between groups).

*** We outline in our response above our justification for using lnCVR in our rat infarct volume dataset. Our paper demonstrates how meta-analyses can be used to quantify heterogeneity in phenotypic outcomes of control and treatment groups with the aim of fostering reproducibility and generalizability of animal models. We therefore agree with the reviewer that it is critical that researchers use the most suitable statistic and model to analyze heterogeneity in reported outcomes (and that CVR is not always appropriate). As we encourage the quantification and embracement of heterogeneity in future studies, we now also address this point on using appropriate heterogeneity statistics based on mean-SD relationships in the main manuscript (line 515-540), so that future analyses of heterogeneity

can be reliably conducted. Finally, to demonstrate the use of alternate methods to analyze heterogeneity, we also now provide a formal, arm-based meta-analysis of lnSD fitting Z-transformed log mean infarct size as a predictor in our supplementary materials (see Fig S4 for lnSD-lnRR galaxy plot and Table S9 for unconditional model estimates). We note that we still identify differences in variability across treatment groups, thus confirming the validity of meta-analyzing variability in treatment outcomes.

3. Is the lower CV with male and females combined just due to larger samples (if we have two identical samples, the CV of the combined sample will be smaller than the CV of the individual samples)?

*** The average N for the combined male/female groups in our control dataset (N_{BOTH} = 7.34) is smaller compared to average N of males (N_{MALE} = 8.88) and is similar to that of females (N_{FEMALE} = 7.31). We therefore do not think that the lower CV estimate in our control group analysis is driven by larger samples.

4. Minor point: 'Reproducibility' has been defined as "re-performing the same analysis with the same code using a different analyst", while the current study is generally discussing what is often termed 'replicability': "re-performing the experiment and collecting new data". Patil, P., Peng, R. D., and Leek, J. (2016). A statistical definition for reproducibility and replicability. bioRxiv. doi: 10.1101/066803

*** We thank the reviewer for this point. We added a small clause in line 244-245 of the Discussion in order to introduce the reader to some of the nuances surrounding the definition of these terms.
