# Supplementary material for: Meta-analysis of variation suggests that embracing variability improves both replicability and generalizability in preclinical research
Source: PLoS Biol. 2021 May 19;19(5):e3001009. doi: 10.1371/journal.pbio.3001009 (PMC8168858; doi:10.1371/journal.pbio.3001009)
Supplement: Attachment — Submitted filename: ResponsetoReviewers.pdf [file pbio.3001009.s022.pdf]

<u>Senior Editor: Roland G Roberts</u>
Based on the reviews, we will probably accept this manuscript for publication, provided you satisfactorily address the remaining points raised by the reviewers. Please also make sure to address the following data and other policy-related requests.

*** We thank Prof Roberts and the Reviewers for their comments and for this decision. We have addressed the remaining points raised and now present the fully revised manuscript.

IMPORTANT: Please attend to the following;
a) Please could you make your title more declarative and informative? We suggest "Meta-analysis of variation in preclinical research suggests that embracing variability would improve both reproducibility and generalizability."

*** We thank Prof Roberts for the suggestion. In order to be more declarative and informative, we have now changed the title of our manuscript to: "Meta-analysis of variation suggests that embracing variability improves both replicability and generalizability in preclinical research"

b) Please attend to the remaining requests from reviewer #2. If you agree with her/his comment about reproducibility vs replicability, please also propagate this change to the title.

*** We have decided to use the term "replicability" instead. We were using reproducibility to specifically refer to "Results reproducibility", which is to obtain the same results of a prior study using the same procedures. This is synonymous to the term replicability (Goodman, Fanelli & Ioannidis 2016 *Sci Transl Med*, 341, 96–102).

c) Please address my Data Policy requests further down. Specifically, please supply numerical values underlying Figs Figs 1, 2AB, 3, S1, S2, S4, S5, S6AB, S7, and cite the location of the data clearly in each relevant Fig legend.

*** We have now addressed the Data Policy requests for publication. All underlying data and code have been deposited in Figshare and addressed in the Figure legends accordingly.

<u>Reviewer #1:</u>
I reviewed the revised manuscript and the responses from the authors. They addressed all of my concerns and I think the manuscript is much improved. I think the paper is now acceptable for publication.

*** We thank the Reviewer for their insightful comments in the last round of review that improved this manuscript.

<u>Reviewer #2:</u>
Please add the p value for the slope of the lnCV-lnH meta regression to the main text

*** Thank you, this has been added.

Also unless it the meanings are different in this field shouldn't all instances of 'reproducibility' be changed to 'replicability' - they can be taken to mean quite different things

*** We thank the reviewer for flagging this point. We have decided to use the term “replicability” instead. We were originally using reproducibility to specifically refer to “Results reproducibility”, which is to obtain the same results of a prior study using the same procedures. This is synonymous to the term replicability (Goodman, Fanelli & Ioannidis 2016 *Sci Transl Med*, 341, 96–102).
